# Ticks (Acari: Ixodidae) parasitizing migrating and local breeding birds in Finland

**DOI:** 10.1007/s10493-021-00679-3

**Published:** 2021-11-17

**Authors:** Jani J. Sormunen, Tero Klemola, Eero J. Vesterinen

**Affiliations:** 1 Biodiversity Unit, University of Turku, Turku, Finland; 2 Department of Biology, University of Turku, Turku, Finland; 3 Department of Ecology, Swedish University of Agricultural Sciences, Uppsala, Sweden

**Keywords:** Birds, *Ixodes ricinus*, *Hyalomma marginatum*, *Ixodes uriae*, *Ixodes lividus*, *Ixodes arboricola*, Ticks, Parasitization, Tick load, Migration

## Abstract

Ticks are globally renowned vectors for numerous zoonoses, and birds have been identified as important hosts for several species of hard ticks (Acari: Ixodidae) and tick-borne pathogens. Many European bird species overwinter in Africa and Western Asia, consequently migrating back to breeding grounds in Europe in the spring. During these spring migrations, birds may transport exotic tick species (and associated pathogens) to areas outside their typical distribution ranges. In Finland, very few studies have been conducted regarding ticks parasitizing migrating or local birds, and existing data are outdated, likely not reflecting the current situation. Consequently, in 2018, we asked volunteer bird ringers to collect ticks from migrating and local birds, to update current knowledge on ticks found parasitizing birds in Finland. In total 430 ticks were collected from 193 birds belonging to 32 species, caught for ringing between 2018 and 2020. Furthermore, four *Ixodes uriae* were collected from two roosting islets of sea birds in 2016 and 2020. Ticks collected on birds consisted of: *Ixodes ricinus* (*n* = 421), *Ixodes arboricola* (4), *Ixodes lividus* (2) and *Hyalomma marginatum* (3). *Ixodes ricinus* loads (nymphs and larvae) were highest on thrushes (Passeriformes: Turdidae) and European robins (*Erithacus rubecula*). The only clearly imported exotic tick species was *H. marginatum*. This study forms the second report of both *I. uriae* and *I. arboricola* from Finland, and possibly the northernmost observation of *I. arboricola* from Europe. The importation of exotic tick species by migrating birds seems a rare occurrence, as over 97% of all ticks collected from birds arriving in Finland during their spring migrations were *I. ricinus*, a species native to and abundant in Finland.

**Supplementary Information:**

The online version contains supplementary material available at 10.1007/s10493-021-00679-3.

## Introduction

Hard ticks (Acari: Ixodidae) are a cosmopolitan family of ectoparasites of terrestrial vertebrates, comprised of approximately 700 species (Guglielmone et al. 2010). Some of these tick species have specific hosts or host animal groups from which they rarely deviate, such as sand martins (*Riparia riparia*) for the sand martin tick (*Ixodes lividus*) or voles for the vole tick (*Ixodes trianguliceps*) (Estrada-Peña et al. 2018; Hillyard 1996). Other species are generalists, feeding on a variety of possible hosts. For example, the sheep tick (*Ixodes ricinus*) and taiga tick (*Ixodes persulcatus*)*,* two of the most common tick species in Eurasia, have both been reported from nearly 300 vertebrate species (Bowman and Nuttall 2008; Uspensky 2016). Included among these species are humans, who serve as incidental, dead-end hosts for the ticks. Unfortunately, these incidental bites frequently result in tick-borne pathogens getting transmitted to humans, who subsequently develop diseases. For example, over 200,000 cases of tick-borne Lyme borreliosis are diagnosed in Europe each year (Marques et al. 2021).

Birds, particularly species that predominately forage on the ground floor, such as thrushes (Passeriformes: Turdidae) and common pheasants (*Phasianus colchicus*), have been identified as important hosts for *I. ricinus* (Buczek et al. 2020). As evidence of frequent contact between birds and ticks, the enzootic cycles of certain frequently detected pathogens have evolved to almost exclusively include birds as reservoirs (Kurtenbach et al. 2002; Norte et al. 2013). Furthermore, several European studies have observed ticks on birds caught for ringing, most often during their spring migrations to nesting areas (Elfving et al. 2010; Hasle 2013; Hasle et al. 2011; Klitgaard et al. 2019; Movila et al. 2013; Sandelin et al. 2015; Toma et al. 2021). This preference for spring migrators as study subjects in Europe is likely due to the added interest provided by possible exotic tick species (and associated pathogens) that are transported in unknown, but likely vast quantities from Africa and Western Asia by the millions of migrating birds. For example, several exotic tick species native to Africa have been reported from migrating birds in studies conducted in southern Italy (Toma et al. 2021). However, ticks from Africa or Western Asia seldom stay attached to birds for long enough to reach the northern parts of Europe, but rather detach from their bird hosts in stopover areas in southern and central Europe. Consequently, in northern Europe, the expected and observed exotic species are mostly those inhabiting the southern and central parts of Europe, rather than those native to Africa or Western Asia (Capligina et al. 2014; Ciebiera et al. 2019; Elfving et al. 2010; Geller et al. 2013; Hasle et al. 2011; Nuorteva and Hoogstraal 1963).

In Finland, only a few studies focusing on ticks found on migrating or local birds have been conducted (Laakkonen et al. 2009; Nuorteva and Hoogstraal 1963; Saikku et al. 1971; Ulmanen et al. 1977). A total of four tick species have been reported in these studies: *I. ricinus*, *I. arboricola*, *I. lividus* and *Hyalomma marginatum*. A paper reporting the first detection of *I. frontalis* from a migrating bird in Finland was published in 2009 (Laakkonen et al. 2009), but the species identification was later rebutted in a commentary to the paper (Heylen et al. 2012). In addition to the four verified species, Jaenson et al. (1994) mention that *I. uriae* has been reported from Finland. Likewise, Estrada-Pena et al. (2018) display a record of the species from the Vaasa region in Finland in their distribution map for the species.

Most of the records concerning ticks found parasitizing birds in Finland date back approximately half a century. Consequently, they are likely no longer representative of the current situation. Due to climate change and the degradation of natural environments by humans, relatively rapid changes are occurring in the geographical distributions of tick species and the availability of habitats suitable for tick survival (Alkishe et al. 2017; Medlock et al. 2013). For example, the survival and development to the adult stage of imported *H. marginatum* was recently reported for the first time in Sweden and England, following the heat wave of the summer of 2018 (Grandi et al. 2020; McGinley et al. 2021). *Hyalomma marginatum* is one of the tick species of interest regarding increasing range size, as it is the main vector for Crimean-Congo Hemorrhagic Fever virus (CCHFV) in Europe (Maltezou et al. 2010). The species is commonly transported to Central and Northern Europe by migrating birds from Africa, Western Asia, and Southern Europe, where the species is endemic (Capek et al. 2014; Nuorteva and Hoogstraal 1963). In addition to the surveillance needed to assess these potential threats from imported ticks and tick-borne pathogens, studies focusing specifically on local birds are needed to produce updated data on the occurrence of bird-associated endophilic tick species. With the data presently available from Finland, there is no way to assess the current distributions or occurrence of such species—let alone their potential impact in, for example, pathogen maintenance.

In 2018, we contacted Finnish bird ringers in order to conduct a study on ticks being imported to Finland by birds during their spring migration. Likewise, we sought to study the diversity of tick species and mean tick loads in migrating and local birds. Here, we present data on the occurrence and species diversity of ticks parasitizing birds in Finland. Likewise, we assess differences between migrating and local birds in mean tick loads and the probabilities of parasitized birds carrying larvae.

## Materials and methods

In February 2018, kits containing sample tubes filled with RNAlater (Thermo-Fisher Scientific, AM7020), tweezers to remove ticks, an information leaflet, and a spreadsheet for recording data were distributed to volunteer bird ringers at the annual national Ringer’s Meeting (Rengastajakokous) held in Seinäjoki, central Finland. In addition, similar kits were distributed to volunteer bird ringers working at, or otherwise affiliated with, the University of Turku. Included in the kits was also a prepaid envelope for sending the samples to the Zoological Museum at the University of Turku (ZMUT). Ticks sent to ZMUT were identified under stereo microscope to species and life stage using morphological keys (Estrada-Peña et al. 2018; Hillyard 1996; Morel and Perez 1973; Nosek and Sixl 1972). Following identification, all samples were placed individually in Eppendorf tubes and stored in a deep freezer at −80 °C to await further analysis.

**Fig. 1:**
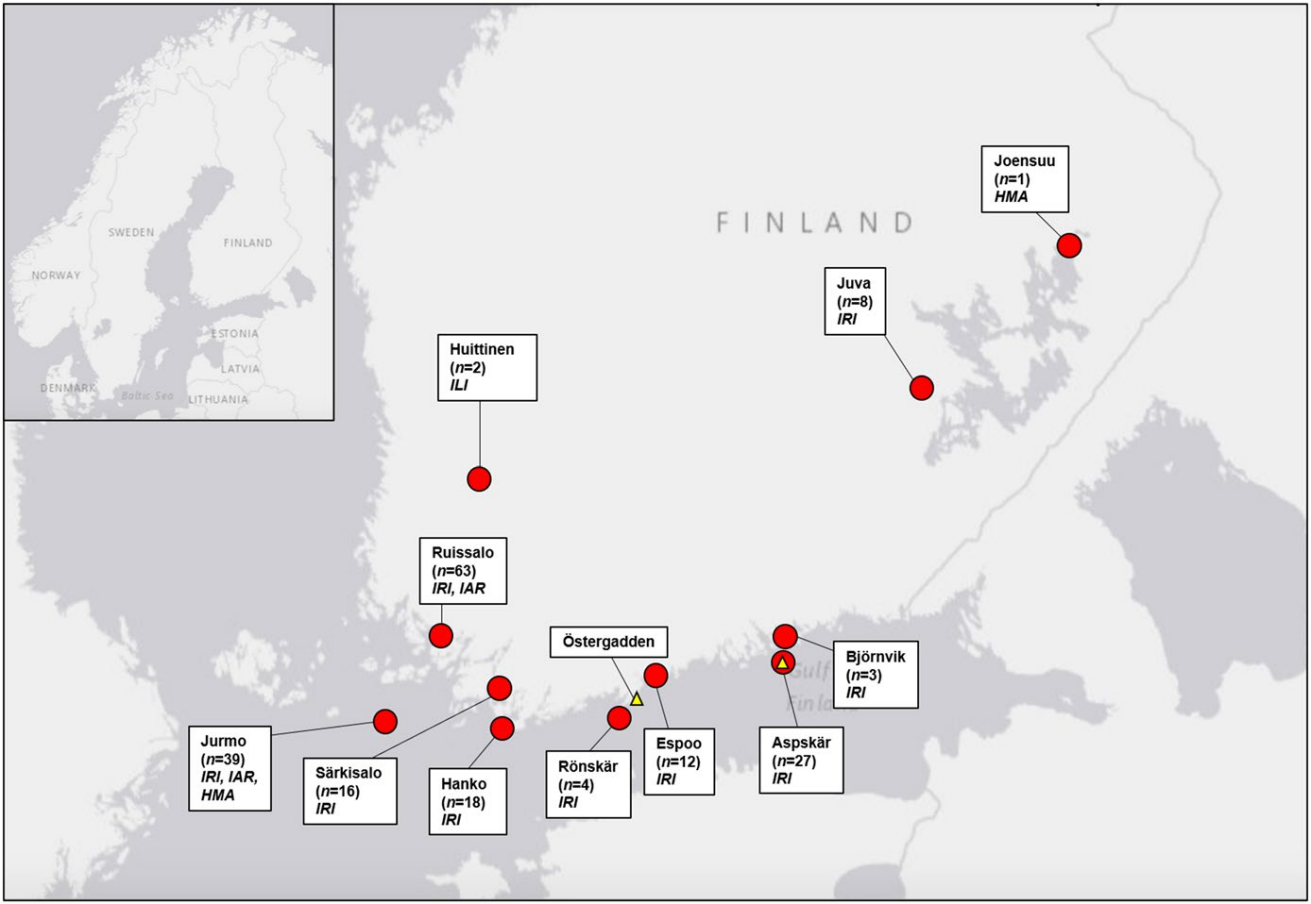
Bird ringing locations. Numbers in parentheses represent the number of birds carrying ticks found from corresponding locations. Tick species found from birds are reported in abbreviated italics (*IRI* *Ixodes ricinus*, *IAR* *I. arboricola*, *ILI* *I. lividus*, *HMA* *Hyalomma marginatum*). Yellow triangles represent locations from where *I. uriae* were found. (Color figure online)

Ringed birds that were parasitized by ticks were divided into three migration categories: spring migrators, residents and autumn migrators. Members of bird species known not to overwinter in Finland that were captured for ringing in March, April or May were classified as spring migrators. Likewise, members of partially migratory species that were freshly ringed between March and early June at two bird observatories on remote islands in the Baltic Sea (Jurmo and Aspskär; Fig. 1) were classified as spring migrators. Birds known to nest in Finland that were ringed between June and September were considered local residents. Finally, individuals ringed in September or October outside the species’ nesting range or on the islands of Jurmo or Aspskär were considered likely autumn migrators.

### Statistical analysis

Differences in mean *I. ricinus* loads between spring migrators and local residents for European robins (*Erithacus rubecula*) and thrushes (*T. merula*, *T. philomelos*, *T. pilaris* and *T. iliacus* collectively) were modelled by generalized linear models (GLM), with negative binomial distribution and log link function. Likewise, differences in the probabilities of spring migrators and local residents of these species/groups carrying larvae were modelled by generalized linear models (GLM) with binary error distribution and logit link function. As nearly all birds carried nymphs and none carried adults, we only analyzed the probability of birds carrying larvae. Too few individuals from these species/groups classified as autumn migrators were available to include them in the analyses. All the GLMs were run with the GLIMMIX procedure of SAS v.9.4. using maximum likelihood estimation.

As we had no data regarding the numbers of birds ringed without ticks, analyses requiring such information were not pursued (such as percentage of birds carrying ticks). Likewise, we refrained from statistical analyses regarding tick load and probability of larval infestation for specific bird species apart from *E. rubecula* and thrushes (collectively), as too few samples, that were also unevenly divided into different migration categories, existed.

## Results

Sample tubes containing ticks collected from a total of 193 birds belonging to 32 species were sent to ZMUT between 2018 and 2020. In addition, four *I. uriae* collected from rocky islets in 2016 and 2020 were delivered to ZMUT and included in the data set. In total, these samples contained 434 ticks, with the following species composition: *I. ricinus* (n = 421), *I. arboricola* (4), *I. uriae* (4), *H. marginatum* (3), and *I. lividus* (2). Out of the birds carrying ticks, 72 were classified as spring migrators, carrying 127 *I.* ricinus and 3 *H. marginatum*. In total 101 birds were classified as local residents, carrying 248 *I. ricinus*, 2 *I. arboricola* and 2 *I. lividus*. Finally, 20 birds were classified as autumn migrators, carrying 46 *I. ricinus* and 2 *I. arboricola*. Species diversity of parasitized birds was highest in local birds (23 species), followed by spring migrators (21) and autumn migrators (7).

Altogether 29 bird species carried *I. ricinus* (Table 1, Tables S1–S3). No adult *I. ricinus* were observed on birds. Table 1 displays data for bird species for which at least four individuals were available regarding total, nymph and/or larvae loads, whereas Tables S1–S3 display the full data by bird species, divided by migration category. Blackbirds (*T. merula*) and European robins (*E. rubecula*) were the species most frequently parasitized by *I. ricinus* (Table 1). Tick loads (total, nymphs, and larvae) were highest on thrushes (*T. merula*, *T. philomelos* and *T. pilaris*; Table 1). Simultaneous detections of both life stages were more common in local birds (22.5% of birds) than in spring or autumn migrators (5.8 and 10.5%, respectively).

**Table 1:** Mean (± SE) total, nymph and larva loads on birds

Bird species	No. infested birds	No. ticks	Mean total tick load	No. birds infested with nymphs	No. nymphs	Mean nymph load	No. birds infested with larvae	No. larvae	Mean larva load
*Carduelis chloris*	8	10	1.3 ± 0.2	7	9	1.3 ± 0.2	–	–	–
*Erithacus rubecula*	31	57	1.8 ± 0.2	23	35	1.5 ± 0.2	11	22	2 ± 0.2
*Fringilla coelebs*	7	12	1.7 ± 0.6	6	7	1.2 ± 0.2	–	–	–
*Parus major*	19	25	1.3 ± 0.2	13	15	1.2 ± 0.1	8	10	1.3 ± 0.2
*Phoenicurus phoenicurus*	15	19	1.3 ± 0.1	10	11	1.1 ± 0.1	6	8	1.3 ± 0.2
*Phylloscopus trochilus*	8	12	1.5 ± 0.4	6	10	1.7 ± 0.5	–	–	–
*Sylvia atricapilla*	6	9	1.5 ± 0.2	–	–	–	4	5	1.3 ± 0.3
*Sylvia borin*	4	4	1 ± 0	–	–	–	–	–	–
*Sylvia communis*	9	22	2.4 ± 0.7	7	9	1.3 ± 0.3	–	–	–
*Sylvia curruca*	8	8	1 ± 0	8	8	1 ± 0	–	–	–
*Turdus merula*	30	94	3.1 ± 0.6	29	71	2.4 ± 0.6	10	23	2.3 ± 0.7
*Turdus philomelos*	10	36	3.6 ± 2.1	10	26	2.6 ± 1.3	–	–	–
*Turdus pilaris*	8	58	7.3 ± 2.6	8	44	5.5 ± 1.9	4	14	3.5 ± 1.7

Data only reported when at least four samples were available

In addition to the bird species parasitized by *I. ricinus*, two sand martins (*R. riparia*) were found with one adult *I. lividus* each. Furthermore, one barred warbler (*Sylvia nisoria*) was parasitized by two *H. marginatum* nymphs, and a wood warbler (*Phylloscopus sibilatrix*) by one. Both birds carrying *H. marginatum* were spring migrators. Finally, two great tits (*Parus major*) carried in total three nymphs and one larva of *I. arboricola*.

No statistically significant differences in mean tick loads (nymphs, larvae or total) were observed between (spring) migrating and local *E. rubecula* or thrushes (Table S4). However, for thrushes, local birds seemed to a have higher probability of carrying larvae than spring migrators [model-derived estimated marginal means and 95% confidence limits: 0.47 (0.31–0.64) vs. 0.14 (0.03–0.44)]. A similar trend (0.63 vs. 0.25) was observed for *E. rubecula*, although due to low sample size, confidence intervals remained wide (Table S4).

## Discussion

The current study reveals that most ticks found from both migrating and resident birds in Finland are *I. ricinus*. This finding conforms to observations of *I. ricinus* being by far the most common tick species detected on migrating birds in northern Europe, with proportions upwards of 90% commonly observed (Capligina et al. 2014; Ciebiera et al. 2019; Geller et al. 2013; Heylen et al. 2017; Kjelland et al. 2010). As an interesting sidenote, no *I. persulcatus* were found from birds, despite them being relatively common in Finland (Laaksonen et al. 2017). However, this may be explained by two points. Firstly, spring migrators arriving from the south have very few locations from which to acquire *I. persulcatus* on the way to Finland (namely Estonia and Latvia) (Capligina et al. 2020; Katargina et al. 2015). Secondly, regarding local birds, ringing was mostly conducted in the southern parts of Finland, where established populations of *I. persulcatus* have not been reported (Laaksonen et al. 2017)—see, however, Zakham et al. (2021) regarding a recent detection of *I. persulcatus* near Helsinki in southern Finland. As this species is known to parasitize birds, it likely can be found on birds during its local activity period in April-June in areas where established populations are present (Pakanen et al. 2020; Sormunen et al. 2020).

Regarding *I. ricinus*, both the species of birds parasitized as well as their tick loads were comparable to those reported in other similar studies from Northern Europe (Capligina et al. 2014; Elfving et al. 2010; Hasle et al. 2011; Kazarina et al. 2015; Kjelland et al. 2010). Thrushes and *E. rubecula* generally had the highest mean *I. ricinus* loads in both spring migrators and local residents, as was also observed in the above listed research papers from the Nordic and Baltic countries. This is generally thought to be due to the ground-foraging behavior of these species, which commonly brings them into contact with juvenile ticks residing in ground floor vegetation and litter (Buczek et al. 2020). Altogether, *I. ricinus* nymphs and larvae were found from 29 different bird species. No *I. ricinus* adults were found from the sampled birds, which is also in line with other similar studies (Buczek et al. 2020).

No obvious differences were observed between migrating and local *E. rubecula* or thrushes in mean tick loads. Consequently, this would suggest that the birds encounter similar numbers of nymphs and larvae in stopover areas during migration as they do at nesting sites in Finland. However, local thrushes appeared to have a higher probability of being parasitized by larvae than spring migrators, indicating a higher frequency of contacts with tick larvae. A similar trend was observed for *E. rubecula*, but the statistical analysis was hampered by the limited sample size (wide confidence intervals). There could be several explanations for this phenomenon. For example, many bird species make their migratory stopovers on their way to Northern Europe in early-mid spring, which may mean that larvae activity at the stopover areas (or source areas for short-distance migrators) is still low. Likewise, foraging habitats may be different in stopover and nesting areas, leading to different rates of contact with tick larvae. It is also possible that the migration flights themselves increase larvae mortality on the host, them being the life stage most susceptible to environmental stresses. However, an equally likely possibility is that this observation is just due to the fact that migrating and local birds have foraged in different locations. The distribution of *I. ricinus* larvae in the nature is known to be very clustered (Nilsson and Lundqvist 1978), so differences in the frequency of larvae parasitization may just signify differences in larvae occurrence between areas.

### Imported exotic tick species

The only clearly imported ticks detected were three *H. marginatum* nymphs. They were removed from a wood warbler (*P. sibilatrix*) and a barred warbler (*S. nisoria*) ringed in May 2018. *Hyalomma marginatum* is a species known to be imported from Southern and Central Europe annually to Northern Europe, and has previously been reported from migrating birds in Finland (Nuorteva and Hoogstraal 1963). The species is particularly prone to travelling vast distances on birds due to its two-host life cycle, wherein the larval and nymphal stages acquire their blood meals from the same host (Hillyard 1996). As such, they spend longer on the host than three-host tick species, which in turn allows for longer travel distances during migration, should the host be a bird. However, the colder summers in the north typically do not allow for the development of immigrant nymphs to the adult stage [however, see Grandi et al. (2020) for an exception], consequently leading to no established populations of the species having been discovered in the Nordic or Baltic countries. Nevertheless, it is possible that the warming climate will, in the future, allow the establishment of populations further north as well (Grandi et al. 2020). In addition, *H. marginatum* serve as vectors for CCHFV, which they can transport to novel areas (Estrada‐Peña et al. 2011; Palomar et al. 2016). The virus has also been reported from *I. ricinus*, whose vector competence remains unclear (Albayrak et al. 2010; Gergova et al. 2012). Consequently, even if the tick species cannot survive at these northern latitudes, it is possible that the virus transported by the tick may establish itself in the local host and/or tick population, highlighting the importance of proper surveillance of migrating birds.

### New records of rarely observed resident species

Regarding rarely documented species that likely have established populations in Finland, three species were encountered during this study: the sand martin tick (*I. lividus*), the sea bird tick (*I. uriae*), and the tree-hole tick (*I. arboricola*).

*Ixodes lividus* is an endophilic tick species that mainly parasitizes sand martins (*R. riparia*) (Hillyard 1996). Previous records of this species from Finland are from two research papers published roughly 50 years previously (Nuorteva and Hoogstraal 1963; Ulmanen et al. 1977). Although two individuals are too few to determine whether an established population exists in Huittinen, they were collected from juvenile birds that were ringed in July, implying that the ticks had attached to the birds in their nests at Huittinen. The role this species might have in the maintenance of pathogens is uncertain (Hillyard 1996). Although they have been reported from some bird species other than sand martins as well (Hillyard 1996), their relatively strict adherence to this particular host and its nests likely renders them insignificant regarding the maintenance of pathogens of medical or veterinary interest.

Another seldom reported species detected is the sea bird tick, *I. uriae*. The only previous report of this species in Finland is from near Vaasa in western Finland (Estrada-Peña et al. 2018). Between 2016 and 2020, a total of four specimens were delivered to ZMUT, three in 2016 from Kirkkonummi (leg. E. Helve) and one in 2020 from near Aspskär, forming the second and third records of the species from the country, respectively (Fig. 1). These specimens were not found attached to birds. Rather, the three *I. uriae* from Kirkkonummi were found from a funneling sample of nest material collected from a great cormorant (*Phalacrocorax carbo*) roost. The tick from Aspskär was found on a bird ringer during a visit to a common murre (*Uria aalge*) roosting islet. Furthermore, in 2018 at the national Ringer’s Meeting, a bird ringer reported brushing off a tick from his arm after a painful bite while ringing seagulls on a rocky islet. This would comply with previous reports of uncharacteristically painful tick bites by *I. uriae* (Hillyard 1996). As the habitats of this species are rarely visited by people other than bird ringers, populations may remain undetected and unreported for untold years. Consequently, specific studies are required to determine the distribution and abundance of *I. uriae* in Finland, as well as to assess their potential role in the local circulation of Lyme borreliosis spirochetes, which the species is known to be able to vector (Duneau et al. 2008). This is particularly current now, as the first report of a successful roosting colony of *P. carbo* in an inland lake in Finland was reported in 2020 (YLE 2020), potentially enabling the spread of the tick to inland freshwater areas.

Finally, *I. arboricola* was detected from great tits (*P. major*) ringed in October 2019. Due to the late date of these detections, it seems unlikely that they could be exported ticks from more southern parts of Europe. Rather, these appear to be individuals that attached themselves to the birds in Finland. Consequently, the finding of two ticks from a great tit from Ruissalo possibly forms the northernmost observation of *I. arboricola* from Europe. However, this status remains somewhat tentative, as the species has been reported on a municipality level from Uppsala, Sweden, the northernmost parts of which reach latitudes further north than Ruissalo (Jaenson et al. 1994). In either case, this study forms the second report of the species from Finland (Saikku et al. 1971). *Ixodes arboricola* is an endophilic tick species mainly parasitizing passerines and some other tree-hole dwelling bird species (Hillyard 1996). As is evident from several reports, including the current study, generalist tick species such as *I. ricinus* are also frequently in contact with passerines. Consequently, *I. arboricola* may have a role in the enzootic cycles of tick-borne pathogens of medical interest. As with *I. lividus* and *I. uriae*, studies specifically designed to assess the distribution and abundance of *I. arboricola* are required to determine the current extent of their occurrence in Finland.

## Conclusions

According to the current study, the importation of exotic tick species to Finland by migrating birds is a rare occurrence. In fact, > 97% of all ticks collected from birds migrating northwards in the spring were *I. ricinus*, a species native to and abundant in Finland. However, despite the low rate of exotic ticks in the migrating birds, the high number of migrating bird individuals may mean that transportation still occurs relatively commonly. It has been estimated that there are about 200–300 million birds in Finland during the summer, of which 90% are short- or long-distance migrants. Furthermore, the importation of foreign members of a native species may also be of consequence, as, in addition to gene flow, novel pathogens may be transported and introduced, or already present pathogens transported to novel areas. More extensive tick collections and analysis of tick-borne pathogens are required to form a more comprehensive picture of the importation of ticks and tick-borne pathogens to Finland. Finally, this study highlights the need for studies specifically targeted at native endophilic tick species parasitizing birds in Finland, for which data are nearly non-existent and outdated.

## Supplementary Information

Below is the link to the electronic supplementary material.

Supplementary file1 (DOCX 24 kb)
