# Supplementary material for: Ticks (Acari: Ixodidae) parasitizing migrating and local breeding birds in Finland
Source: Exp Appl Acarol. 2021 Nov 17;86(1):145–56. doi: 10.1007/s10493-021-00679-3 (PMC8702513; doi:10.1007/s10493-021-00679-3)
Supplement: Supplementary file 1 — Supplementary file1 (DOCX 24 kb) [file 10493_2021_679_MOESM1_ESM.docx]

Table S1. Birds infested with *Ixodes ricinus* ticks caught for ringing during spring migration and associated tick loads.

| Species | Number of birds infested with nymphs | Nymphs | Mean nymph load (±SE) | Number of birds infested with larvae | Larvae | Mean larvae load | Bird individuals in Finland (Red Book 2019) |
| --- | --- | --- | --- | --- | --- | --- | --- |
| *Acrocephalus dumetorum* | 1 | 1 | 1 ± 0 | - | - | - | 85 000 |
| *Acrocephalus palustris* | 1 | 2 | 1 ± 0 | - | - | - | 41 000 |
| *Anthus trivialis* | 1 | 4 | 4 ± 0 | - | - | - | 3,5 million |
| *Carduelis chloris* | 1 | 2 | 2 ± 0 | - | - | - | 580 000 |
| *Carduelis spinus* | - | - | - | 1 | 1 | 1 ± 0 | 3,3 million |
| *Erithacus rubecula* | 10 | 17 | 1.7 ± 0.4 | 3 | 6 | 2 ± 0.6 | 4,6 million |
| *Fringilla coelebs* | 3 | 4 | 1.3 ± 0.3 | - | - | - | 15 million |
| *Luscinia luscinia* | - | - | - | 1 | 1 | 1 ± 0 | 42 000 |
| *Luscinia svecica* | 1 | 1 | 1 ± 0 | - | - | - | 110 000 |
| *Parus major* | - | - | - | 1 | 1 | 1 ± 0 | 3,7 million |
| *Phoenicurus phoenicurus* | 10 | 11 | 1.1 ± 0.1 | 6 | 8 | 1.3 ± 0.2 | 1,2 million |
| *Phylloscopus trochilus* | 4 | 5 | 1.3 ± 0.3 | 2 | 2 | 1 ± 0 | 14 million |
| *Prunella modularis* | 1 | 1 | 1 ± 0 | - | - | - | 970 000 |
| *Sylvia atricapilla* | 1 | 2 | 2 ± 0 | 1 | 1 | 1 ± 0 | 220 000 |
| *Sylvia borin* | 2 | 2 | 1 ± 0 | - | - | - | 1,7 million |
| *Sylcia communis* | 1 | 1 | 1 ± 0 | - | - | - | #N/A |
| *Sylvia curruca* | 5 | 5 | 1 ± 0 | - | - | - | 590 000 |
| *Turdus merula* | 9 | 19 | 2.1 ± 0.6 | - | - | - | 1,2 million |
| *Turdus philomelos* | 5 | 20 | 4 ± 2.5 | 2 | 9 | 4.5 ± 3.5 | 2 million |
| TOTAL | 56 | 97 | 1.7 ± 0.3 | 17 | 29 | 1.7 ± 0.4 |  |

Table S2. Local birds infested with *Ixodes ricinus* ticks caught for ringing and associated tick loads.

| Species | Number of birds infested with nymphs | Nymphs | Mean infestation intensity (±SE) | Number of birds infested with larvae | Larvae | Mean infestation intensity (±SE) | Bird individuals in Finland (Red Book 2019) |
| --- | --- | --- | --- | --- | --- | --- | --- |
| *Acrocephalus dumetorum* | 1 | 1 | 1 ± 0 | - | - | - | 85 000 |
| *Acrocephalus schoenobaenus* | 2 | 2 | 1 ± 0 | - | - | - | 210 000 |
| *Anthus trivialis* | 1 | 1 | 1 ± 0 | - | - | - | 3,5 million |
| *Carduelis chloris* | 6 | 7 | 1.2 ± 0.2 | 1 | 1 | 1 ± 0 | 580 000 |
| *Parus caeralus* | 1 | 1 | 1 ± 0 | - | - | - | #N/A |
| *Emberiza schoeniclus* | 1 | 1 | 1 ± 0 | - | - | - | 480 000 |
| *Erithacus rubecula* | 5 | 7 | 1.4 ± 0.2 | 5 | 9 | 1.8 ± 0.4 | 4,6 million |
| *Ficedula hypoleuca* | 1 | 1 | 1 ± 0 | 1 | 2 | 2 ± 0 | 1 million |
| *Fringilla coelebs* | 3 | 3 | 1 ± 0 | 2 | 5 | 2.5 ± 1.5 | 15 million |
| *Fringilla montifringilla* | 2 | 2 | 1 ± 0 | - | - | - | 2,6 million |
| *Motacilla alba* | 1 | 1 | 1 ± 0 | - | - | - | 990 000 |
| *Parus major* | 11 | 12 | 1.1 ± 0.1 | 6 | 7 | 1.2 ± 0.2 | 3,7 million |
| *Parus montanus* | 1 | 1 | 1 ± 0 | - | - | - | #N/A |
| *Phylloscopus trochilus* | 2 | 5 | 2.5 ± 1.5 | - | - | - | 14 million |
| *Sylvia atricapilla* | 2 | 2 | 1 ± 0 | 3 | 4 | 1.3 ± 0.3 | 220 000 |
| *Sylvia borin* | 1 | 1 | 1 ± 0 | 1 | 1 | 1 ± 0 | 1,7 million |
| *Sylvia communis* | 6 | 8 | 1.3 ± 0.3 | 3 | 13 | 4.3 ± 0.9 | 700 000 |
| *Sylvia curruca* | 2 | 2 | 1 ± 0 | - | - | - | 590 000 |
| *Turdus iliacus* | 3 | 14 | 4.3 ± 1.8 | 1 | 1 | 1 ± 0 | 2,8 million |
| *Turdus merula* | 18 | 47 | 2.6 ± 0.9 | 10 | 23 | 2.3 ± 0.7 | 1,2 million |
| *Turdus philomelos* | 4 | 5 | 1.3 ± 0.3 | 1 | 1 | 1 ± 0 | 2 million |
| *Turdus pilaris* | 8 | 44 | 5.5 ± 1.9 | 4 | 14 | 3.5 ± 1.7 | 3,2 million |
| TOTAL | 82 | 167 | 2.0 ± 0.3 | 38 | 81 | 2.1 ± 0.3 |  |

Table S3. Birds infested with *Ixodes ricinus* ticks caught for ringing during autumn migration and associated tick loads.

| Species | Number of birds infested with nymphs | Nymphs | Mean nymph load (±SE) | Number of birds infested with larvae | Larvae | Mean larvae load  (±SE) | Bird individuals in Finland (Red Book 2019) |
| --- | --- | --- | --- | --- | --- | --- | --- |
| *Emberiza rustica* | 1 | 2 | 2 ± 0 | 0 | - | - | 440 000 |
| *Erithacus rubecula* | 8 | 11 | 1.4 ± 0.2 | 3 | 7 | 2.3 ±0.3 | 4,6 million |
| *Fringilla montifringilla* | 1 | 4 | 4 ± 0 | 1 | 10 | 10 ±0 | 2,6 million |
| *Parus*  *major* | 2 | 3 | 1.5 ± 0.5 | 1 | 2 | 2 ±0 | 3,7 million |
| *Sylvia curruca* | 1 | 1 | 1 ± 0 | 0 | - | - | 590 000 |
| *Turdus merula* | 2 | 5 | 2.5 ± 1.5 | 0 | - | - | 1,2 million |
| *Turdus philomelos* | 1 | 1 | 1 ± 0 | 0 | - | - | 2 million |
| TOTAL | 16 | 27 | 1.7 ± 0.3 | 5 | 19 | 3.8 ± 1.6 |  |

Table S4. Detailed results of statistical analyses regarding tick loads and infestation probability for *Erithacus rubecula* and thrushes (*Turdus merula*, *T. philomerlos*, *T. pilaris, T. iliacus*) between local birds and spring migrators.

| Species^a^ | Response variable | Metrics | Mean estimate^b^ | | 95% CL |
| --- | --- | --- | --- | --- | --- |
| ERIRUB | Nymph load | GLM, *n*=20, F_1, 18_=0.93, *p*=0.35 | LO  SM | 0.91  1.4 | 0.42 – 2.0  0.83 – 2.3 |
| ERIRUB | Larvae load | GLM, *n*=20, F_1, 18_=1.55, *p*=0.23 | LO  SM | 1.1  0.5 | 0.44 – 2.9  0.18 – 1.4 |
| ERIRUB | Total tick load | GLM, *n*=20, F_1, 18_=0.00, *p*=0.96 | LO  SM | 2.0  1.9 | 1.2 – 3.3  1.3 – 3.0 |
| ERIRUB | Probability of larvae infestation | GLM, *n*=20, F_1, 18_=2.65, *p*=0.12 | LO  SM | 0.63  0.25 | 0.26 – 0.89  0.08 – 0.57 |
| Thrushes | Nymph load | GLM, *n*=48, F_1, 46_=0.20, *p*=0.66 | LO  SM | 3.2  2.8 | 0.54 – 2.3  0.74 – 1.6 |
| Thrushes | Larvae load | GLM, *n*=48, F_1, 46_=0.76, *p*=0.39 | LO  SM | 1.1  0.64 | 0.58 – 2.3  0.21 – 2.0 |
| Thrushes | Total tick load | GLM, *n*=48, F_1, 46_=0.56, *p*=0.46 | LO  SM | 4.4  3.4 | 3.1 – 6.1  2.0 – 5.9 |
| Thrushes | Probability of larvae infestation | GLM, *n*=48, F_1, 46_=4.00, *p*=0.051 | LO  SM | 0.47  0.14 | 0.31 – 0.64  0.03 – 0.44 |

^a^ERIRUB: *Erithacus rubecula;* Thrushes: *Turdus merula*, *T. philomelos*, *T. pilaris*, *T. iliacus*

^b^LO: local birds; SM: spring migrators
